# Selection of thermodynamic models for combinatorial control of multiple transcription factors in early differentiation of embryonic stem cells

**DOI:** 10.1186/1471-2164-9-S1-S18

**Published:** 2008-03-20

**Authors:** Chieh-Chun Chen, Xin-Guang Zhu, Sheng Zhong

**Affiliations:** 1 Department of Bioengineering, University of Illinois at Urbana Champaign, Urbana, IL 61801, USA; 2 Department of Computer Science, University of Illinois at Urbana Champaign, Urbana, IL 61801, USA; 3 Department of Plant Biology, University of Illinois at Urbana Champaign, Urbana, IL 61801, USA; 4 Department of Statistics, University of Illinois at Urbana Champaign, Champaign, IL 61820, USA; 5 Institute of Genomic Biology, University of Illinois at Urbana Champaign, IL 61801, USA

## Abstract

**Background:**

Transcription factors (TFs) have multiple combinatorial forms to regulate the transcription of a target gene. For example, one TF can help another TF to stabilize onto regulatory DNA sequence and the other TF may attract RNA polymerase (RNAP) to start transcription; alternatively, two TFs may both interact with both the DNA sequence and the RNAP. The different forms of TF-TF interaction have different effects on the probability of RNAP's binding onto the promoter sequence and therefore confer different transcriptional efficiencies.

**Results:**

We have developed an analytical method to identify the thermodynamic model that best describes the form of TF-TF interaction among a set of TF interactions for every target gene. In this method, time-course microarray data are used to estimate the steady state concentration of the transcript of a target gene, as well as the relative changes of the active concentration for each TF. These estimated concentrations and changes of concentrations are fed into an inference scheme to identify the most compatible thermodynamic model. Such a model represents a particular way of combinatorial control by multiple TFs on a target gene.

**Conclusions:**

Applying this approach to a time-course microarray dataset of embryonic stem cells, we have inferred five interaction patterns among three regulators, Oct4, Sox2 and Nanog, on ten target genes.

## Background

Quantitative models describing gene expression in terms of quantity, speed and timing in different environmental contexts are essential for the study of many biological processes. Thermodynamic models are based on the assumption that the level of gene expression is proportional to the equilibrium probability that RNA polymerase (RNAP) is bound to the promoter of the interested gene and these probabilities can be computed in a statistical mechanics framework. In prokaryotes under well studied assumptions, a function is available to relate each particular form of interaction among transcription factors (TFs) and RNAP to the level of the expression of the target gene [1-3]. Such functions are termed “regulation factors” [1]. There are to date few discussions on the extent to which these regulation factors hold for eukaryotes [3].

In this paper we propose a method to select regulation factors, i.e. to infer the form of TF-TF and TF-RNAP interactions for each target gene. This method enables the investigation of regulation factors from empirical data in eukaryotic systems. Applying this method to a time course microarray dataset of retinoid acid induced differentiation of mouse embryonic stem cells (ESCs) [4], we clearly distinguish different interaction forms among Oct4, Sox2 and Nanog, and their roles as activators, repressors and helpers on each target gene. The detailed characterization of interaction forms among multiple transcription factors allows us to build a core transcription network in ESCs using a bottom-up approach.

ESCs are derived from early mammalian embryos and can be propagated through apparently unlimited, undifferentiated proliferation (self-renewal) in cultured cell lines (mouse: [5,6], human: [7]). ESCs possess several notable properties that account for their exceptional scientific and medical importance. ESCs have remarkable potential to develop into many different cell types in the body (known as pluripotency [8]) and therefore they may be used to study both normal and abnormal body developments. A major challenge in the study of ESCs is to explain how the complex gene network is wired to control their properties of pluripotency and self-renewal. Transcriptional control is thought to be a key control mechanism for ESCs to maintain their undifferentiated state [9-16]. Regulatory proteins and relevant genomic sequences work together to precisely tune the expression levels of thousands of target genes in ESCs. The interactions among these regulatory proteins and their interactions with particular genomic sequences collectively define a transcription network. Understanding of the part of the network at work in ESCs, i.e. the functional state of the transcription network in ESCs, can reveal how the undifferentiated state of ESCs is maintained, and how it can be disrupted to initiate different routes of differentiation.

## Results

### Simulation data

**Figure 1:**
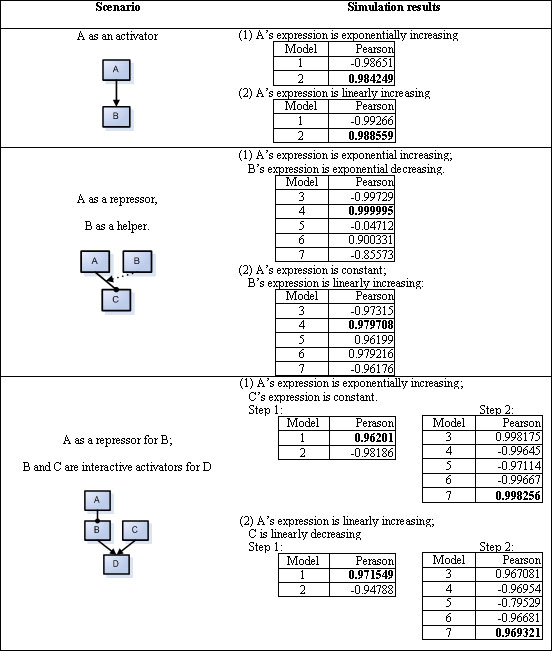
**Results from synthetic data using the Interaction-Identifier algorithm**. The concentration of A is simulated using either a linear function: [TF] = 500+500T, or an exponential function [TF] = 500+200logT, where T is the time in both equations.

We use three regulatory patterns to test our new algorithm (Figure 1). Under the first regulatory pattern (Row 1, Figure 1), we do two simulations . First, TF's expression increases linearly over time: real_TF_exp = 500+500T, where T=2, 4, 8, 16, 32, 64 and 128. In the second simulation the TF's expression increases exponentially over time: real_TF_exp = 500+200logT, where T=2, 4, 8, 16, 32, 64 and 128. Because there is only one TF in consideration, there are only two candidate regulatory models, either repression (Model 1) or activation (Model 2). In both simulations our method correctly picks Model 2 (Row 1, Figure 1). Two simulations are performed under the second regulatory pattern (Row 2, Figure 1). For each simulation, our method consistently identifies the correct regulatory model out of five candidate models (Row 2, Figure 1). Under the third regulatory pattern, we do a two-step analysis. In the first step, we apply the method to identify the regulatory relationship between TFs A and B (Row 3, Figure 1), i.e. one TF controls the expression of another TF. After a regulatory model is determined between A and B, we use the expression pattern of B derived from the Step 1 to identify the interaction form between TFs B and C. There are two candidate models for Step 1 and five candidate models for Step 2. Altogether 10 potential regulatory models exist among the four genes. In two independent simulations, our method has both identified the correct regulatory models (Row 3, Figure 1).

### Sensitivity analysis

We check to what extent the choices of parameters affect the method performance. Regulatory model 7 (the regulatory pattern between B, C, D in Row 3, Figure 1) is chosen to perform the sensitivity analysis. We vary K_TF_, K_g_, K_d_ and q_p_ in wide ranges, for example an 10000 fold range for K_TF_, and re-run our algorithm. Results in Table 1 show that the method can robustly identify the correct regulatory model even if the parameters are varied by 100 fold. The only exceptions are the cases where the synthesis rates of mRNA are set to be too slow – below 1 mRNA molecule per 10 minute, as compared to the default of 10 mRNA per minute from empirical data. We therefore do not suggest using a very small synthesis rate.

**Table 1:** Sensitivity test for K_TF_ , K_g_ , q_p_, K_d_ and H. Numbers in bold represent the highest correlations under each parameter set. The results indicate that the correct model can be identified even with drastic variation in parameters used in the model.

T	K_TF_*	Pearson	Kg	Pearson	Kd	Pearson	q_p_	Pearson	H	Pearson
D	1		10		60/36		0.05		2	
model 3	0.01	0.950042	1/60	0.967087	60/24	0.967081	1/35	0.967742	1	0.971055
	0.1	0.950513	1/6	0.967081	60/30	0.967081	0.05	0.967081	2	0.967081
	1	0.967081	10	0.967081	60/36	0.967081	0.10	0.966161	3	0.963737
	10	0.957063	600	0.967081	60/42	0.967081	1	0.964237	4	0.964052
	100	0.956187	1000	0.967081	60/48	0.967081	10	0.963906	5	0.968799
model 4	0.01	-0.95142	1/60	-0.96956	60/4	-0.96954	1/35	-0.96947	1	-0.97152
	0.1	-0.95166	1/6	-0.96953	60/30	-0.96954	0.05	-0.96954	2	-0.96954
	1	-0.96954	10	-0.96954	60/36	-0.96954	0.10	-0.96969	3	-0.96932
	10	-0.95665	600	-0.96954	60/42	-0.96954	1	-0.97154	4	-0.97191
	100	-0.95622	1000	-0.96954	60/48	-0.96954	10	-0.97186	5	-0.97484
model 5	0.01	-0.88219	1/60	-0.79531	60/24	-0.79529	1/35	-0.79534	1	-0.92423
	0.1	-0.97198	1/6	-0.7953	60/30	-0.79529	0.05	-0.79529	2	-0.79529
	1	-0.79529	10	-0.79529	60/36	-0.79529	0.10	-0.79518	3	-0.59358
	10	-0.61602	600	-0.79529	60/42	-0.79529	1	-0.79363	4	-0.40171
	100	-0.6125	1000	-0.79529	60/48	-0.79529	10	-0.78977	5	-0.2617
model 6	0.01	0	1/60	-0.96681	60/24	-0.96681	1/35	-0.96684	1	-0.96778
	0.1	-0.97196	1/6	-0.9668	60/30	-0.96681	0.05	-0.96681	2	-0.96681
	1	-0.96681	10	-0.96681	60/36	-0.96681	0.10	-0.96674	3	-0.95978
	10	-0.66535	600	-0.96681	60/42	-0.96681	1	-0.96544	4	-0.93539
	100	-0.61378	1000	-0.96681	60/48	-0.96681	10	-0.95794	5	-0.8747
**model 7**	0.01	**0.96079**	1/60	**0.969306**	60/24	**0.969321**	1/35	**0.969576**	1	**0.97157**
	0.1	**0.961556**	1/6	**0.96932**	60/30	**0.969321**	0.05	**0.969321**	2	**0.969321**
	1	**0.969321**	10	**0.969321**	60/36	**0.969321**	0.10	**0.96904**	3	**0.968987**
	10	**0.709205**	600	**0.96932**	60/42	**0.969321**	1	**0.968629**	4	**0.973865**
	100	**0.614337**	1000	**0.96932**	60/48	**0.96932**	10	**0.968572**	5	**0.98013**
model 8	0.01	-0.97205	1/60	-0.92839	60/24	-0.9284	1/35	-0.92828	1	-0.94378
	0.1	-0.97213	1/6	-0.9284	60/30	-0.9284	0.05	-0.9284	2	-0.9284
	1	-0.9284	10	-0.9284	60/36	-0.9284	0.10	-0.92855	3	-0.86111
	10	-0.95055	600	-0.9284	60/42	-0.9284	1	-0.92884	4	-0.73939
	100	**-0.95336**	1000	-0.9284	60/48	-0.9284	10	-0.92888	5	-0.68121
* The unit number of K_TF_ is the maximum expression value /10.										

### Interaction models for Oct4 and Nanog in mouse embryonic stem cells

Oct4, Sox2 and Nanog are the key transcription factors to maintain pluripotency ESCs. Nanog is known to be jointly regulated by Oct4 and Sox2.

**Figure 2:**
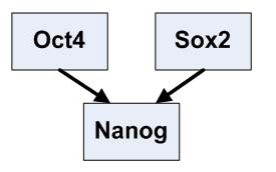
**The identified interation form of Oct4 and Sox2 on Nanog**.

**Figure 3:**
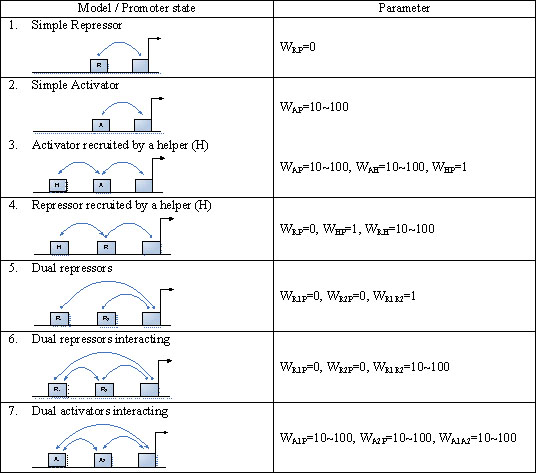
**Forms of TF-RNAP interactions and their corresponding parameters for modeling the probability of RNAP binding**. A_1_ and A_2_ are transcription factors acting as activators of genes. R_1_ and R_2_ are transcription factors acting as repressors of genes. A box without label represents RNAP. A curve with a bar at the end represents a repression effect; a curve with an arrow at the end indicates either cooperation between transcription factors or activation of a gene by a transcription factor.

**Figure 4:**
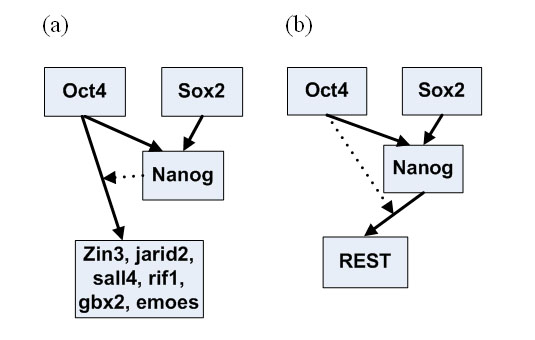
**The different regulatory networks of different groups of target genes identified using Interaction-Identification algorithm**. The directed arrows represent activation and the dotted line represents the function of a helper. The relationship between Nanog and Oct 4 with these target genes follows the model 3 in Figure 3.

**Figure 5:**
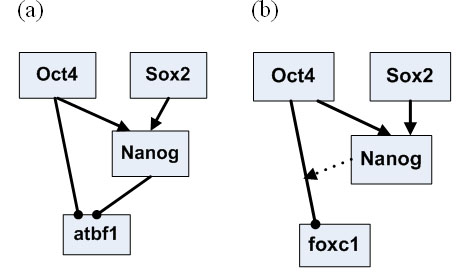
**The different regulatory networks of different groups of target genes identified using the Interaction-Identifier algorithm**. (a) model 5 (Figure 3) (b) model 4 (Figure 3), where the dotted line represents the function of a helper; a line with an arrow at the end represents the effect of an activator; a line with a solid dot at the end represents the effect of a repressor.

Time course microarray data have been generated for retinoid acid induced differentiation of mouse ESCs [4]. Genes that are jointly regulated by Oct4 and Nanog have been reliably identified [13]. Among these target genes, nine genes (Jarid2, Sall4, Rif1, Gbx2, REST, Zin3, Foxc1, Smarcad1 and Atbf1) are represented on the Affymetrix U133 microarray and therefore their time course data are available [4]. We first apply the Interaction-Identifier method to identify the regulatory model for Nanog, following the same procedure as we did for the synthetic data. The time course expression data suggest that Oct4 and Sox2 help each other to stabilize onto the regulatory sequence and attract the RNAP (Figure 2). We then identify the regulatory models for the Oct4 and Nanog regulated genes. Although these nine genes are all regulated by Oct4 and Nanog in ESCs, they are not regulated under the same mechanism. Jarid2, Sall4, Rif1 and Gbx2, are regulated under model 7 (Figure 3), where Oct4 and Nanog are synergistic activators. REST and Zic3 are both regulated under model 3, with one TF being an activator and the other a helper (Figure 4). Atbf1 is regulated under model 5 where Oct4 and Nanog are independent repressors (Figure 5A). Foxc1 is regulated under model 4 where Nanog is a helper and Oct4 is a repressor (Figure 5B). These results suggest that Atbf1 and Foxc1 are probably involved in lineage differentiation and therefore need to be repressed by key transcription factors in ESCs. Indeed, Foxc1 is involved in ocular development [17] and Abf1 mRNA is found to be abundant in prostate [18]. Finally, none of the models being considered derives an expression pattern similar to the observed expression pattern of Smarcad1 (All Pearson correlations are smaller than 0.5). This may suggest that besides Oct4 and Nanog, there are other mechanisms responsible for the transcriptional control of Smarcad1.

## Discussion

New algorithms combining the strengths of both mechanistic modeling and statistical inference approaches to identify genetic regulatory networks are in demand. The methodology proposed in this study is one step towards this goal. This new method integrates three pieces of information together to infer a genetic regulatory network: a) mechanistic models of transcription factor binding and RNA transcription [3], b) prior knowledge of network components based on ChIP-chip data, c) time course expression data. Furthermore, this method combines two methodologies together, kinetic modelling and correlation analysis. In the following, we further compare this new method with previous efforts in the same vein and explain the rationale and assumptions of this new approach.

We choose to represent the expression levels as continuous instead of discrete variables in this study. Reverse engineering approaches have been developed to infer boolean networks underlying changes in the gene expression levels, assuming that expression levels of different genes can be categorized into different states [19]. In reality, gene expression levels reflected by microarray data tend to be continuous rather than discrete. Furthermore, continuous signals have a greater capacity over discrete signals in implementing different control functions, such as signal transformation and transduction, precise feedback and feed forward loops and maintaining homeostasis [20]. An implicit assumption of using continuous concentrations of the chemical species (mRNA and protein) is that the stochastic fluctuations due to single molecules are ignored. In both prokaryotic and eukaryotic cells, noises in gene expression levels have been observed and suggested to be an evolvable trait, which possibly plays a role in cellular phenotypic variation and cellular differentiation [21-24]. Both stochasticity inherent in the biochemical process of gene expression (intrinsic noise) and fluctuations in other cellular components (extrinsic noise) contribute substantially to overall phenotypic variation [21]. In this study, the mRNA signals obtained are effectively averages of pooled populations of cells; where the influence of stochastic noise of single molecules on chemical concentration (mRNA and protein) are presumably effectively decreased.

Three other assumptions are made in the methodological framework. First the form of the interaction among TFs and RNAP are assumed to be invariant for the different conditions under which the gene expression data are obtained. This assumption can be violated when the experimental conditions are dramatically different from each other, for example, under different stress conditions. This assumption is better satisfied by using data from one biological process, for example, a developmental process. For this reason we suggest using time course gene expression data rather than data generated from different experimental conditions. Even for time course data, the users should exercise caution, because the regulation factor can still change in some circumstances, such as when the cell goes through different phases of the cell cycle [25,26]. The second assumption is that the transcriptome is at equilibrium state at each time point when the gene expression is measured. This assumption is satisfied by most of the time course microarray data that the authors are aware of, and users can check this assumption by examining the reproducibility of the data among biological replicates. The third and the biggest assumption is that the thermodynamic models derived and tested for prokaryotes can be applied to eukaryote systems. This is essentially ignoring a number of transcriptional regulatory mechanisms that eukaryotes utilize, such as chromatin modification and enhancer effects. However as an approximation, the Interaction-Identifier method is still useful to analyze the biophysical properties of known TFs. Another point in favor of the validity of this method is that the absolute value of the model-derived gene expression level does not influence the correlation calculation. Only the pattern of change of the expression levels over time influence the correlation calculation. Many of the eukaryotic specific regulatory features, such as the distance between the enhancer and the promoter, are invariant for the target gene over the time course, and therefore such features should not affect the selection of the corrected model.

Previously, models were developed to infer genetic regulatory networks from time series data that are generated before the equilibrium is reached [27,28]. There are, however, a lot more experiments generating gene expression data at steady states in a time series manner. In this paper we demonstrate that steady state time series data can be utilized to effectively characterize the interaction forms among multiple transcription factors. The Interaction-Identifier method should therefore be applicable to analyze a larger number of biological processes where steady state time course data are available. 

## Conclusions

We developed Interaction-Identifier methods for identifying interaction forms of TFs. We applied it to analyze the combinatorial control of the key transcription factors in mouse ESCs. ESCs are pluripotent cells derived from the inner cell mass of the mammalian blastocyst. They are capable of indefinite self-renewing expansion in culture. Depending on culture conditions, these cells can differentiate into a variety of cell types [29]. The ability to steer ESC differentiation into specific cell types holds great promise for regenerative medicine [13,30-32]. A few transcription factors have shown to be key transcriptional regulators in ESCs. These include Oct4, Sox2, Nanog and others [4,13]. Large scale genomic data have been generated for these regulators, including ChIP-PET (a technology close to ChIP-chip) [13] and time course microarray data [4]. Albeit the availability of the high-throughput genomic data, the regulatory circuit in ESC still await quantitative and realistic models to be described. We regard a realistic model for quantifying the effect of combinatorial control of multiple ESC regulators as a firm building block towards understanding the whole network. In this paper we explored Interaction-Identifier method to infer the interaction patterns between multiple ESC regulators. In particular, Interaction-Identifier method identifies that Oct4 and Sox2 help each other to stabilize onto DNA and attract the RNAP. This indicates that the DNA-bound Oct4 will be less in Sox2 knock-down ESCs, and vice versa. This is in line with the fact that the knock-down of either of the two transcription factors will decrease the expression levels of the mutual target genes and start the differentiation process [4]. We have subsequently categorized the mutual targets of Oct4 and Nanog according to the pattern of their combinatorial effect. Although Oct4 and Nanog often serve as activators for maintaining the expression of ESC specific genes, they also inhibit genes for lineage specific differentiation. Little is known about how Oct4 and Nanog switch their tasks between activators and repressors. Among all the identified regulatory patterns, Oct4 and Sox2 generally do not attract RNAP at the same time, but rather one serves as the helper to the other (Figures 4, 5). Only in one case Oct4 and Nanog both interact with RNAP, where both serve as repressors (Figure 5A). This result suggests that when both of the two transcription factors interact with RNAP, they perform an inhibition task.

## Methods

**Figure 6:**
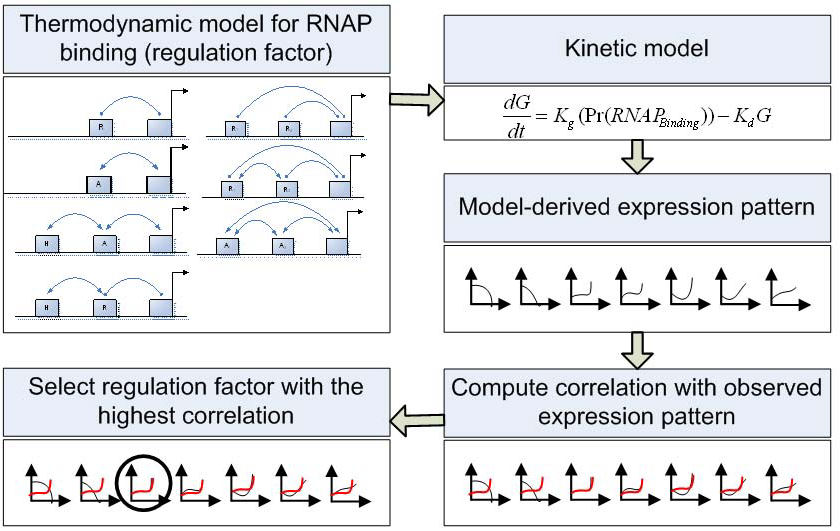
**Flowchart of the Interaction-Identifier algorithm**.

We propose an Interaction-Identifier method to identify the candidate form of interaction among the TFs and RNA polymerase (RNAP) on the promoter of a target gene. This method begins by using a thermodynamic function, termed regulation factor, to predict the equilibrium probability that RNAP binds to the promoter of its targeted gene (P_RNAP_) based on concentrations of associated TFs and interaction forms among TFs and RNAP. Next, it uses systems of ordinary differential equations to simulate the dynamics of expression interested genes, assuming a) the transcription rate is proportional to the P_RNAP_; b) mRNA degradation rate is linearly dependent on the RNA concentration; c) the concentration changes of TF factor can be inferred from the changes in the mRNA levels of TFs. Thirdly, using measured time course gene expression data from microarray experiments, we compute the Pearson correlation coefficient and Euclidean distance between the observed expression pattern and the predicted expression pattern. Since different interaction forms among TFs and RNAP will lead to different regulation factors, Interaction-Identifier method conducts these first three steps for all interaction forms between TFs and promoters. Finally, the regulation factor that predicts an expression pattern with highest correlation to the observed expression pattern is identified as most plausible interaction form that TFs take to regulate this target gene (Figure 6).

### Thermodynamic models for RNAP binding

Cells receive a wide variety of cellular and environmental signals, which are often processed combinatorially to generate specific genetic responses. We follow Buchler et al (2003) to integrate combinatorial signal at the level of cis-regulatory transcription control. Also see Bintu et al for review [1-3]. In this section this theoretical framework [1-3] is briefed.

RNAP binds to promoter of a target gene to initiate gene transcription. The promoter of a given gene can only take one of the two states in a given time in a cell: with or without RNAP binding. We denote the ratio of the probability of promoter bound by RNAP to unbound by RNAP as *q_p_* (Table 2).

**Table 2:** The Bernoulli distribution for the two states of a promoter.

**State**	**RNAP**	**Weight**
1	0	1
2	1	*q_p_*

The weight *q_p_* denotes the ratio between the probabilities of the two states of promoter.

It follows that the percentage of the promoter of the target gene being bound with an RNAP is $P[RNAP_{binding}]=\frac{q_{p}}{1+q_{p}}$.

If we consider there is an TF interacting with RNAP, a promoter can then take one of the four possible states: (1) both the TF and the RNAP bind to the promoter; (2) Only the RNAP binds to the promoter; (3) Only the TF binds to the promoter; (4) neither the TF nor the RNAP binds to the promoter (Table 3).

**Table 3:** The multinomial distribution of a promoter with one TF and its RNAP.

**State**	**TF**	**RNAP**	**Weight**
1	0	0	1
2	0	1	*q_p_*
3	1	0	*q_TF_*
4	1	1	*w_TFp_q_p_q_TF_*

The weights *q_p_*, *q_TF_* and *w_TFp_q_p_q_TF_* denote the ratios between the probabilities of States 2,3,4 respectively assuming the weight of promoter with no RNAP or TF is 1.

Let P_i_ denote the probability of a promoter in the i^th^ state. It follows that the probability of the promoter of the target gene being bound with an RNAP is

$$P[RNAP_{binding}]=\frac{P_{2}+P_{4}}{P_{1}+P_{2}+P_{3}+P_{4}}=\frac{q_{p}+w_{TFp}q_{TF}q_{p}}{1+q_{p}+q_{TF}+w_{TFp}q_{TF}q_{p}}$$

, where $w_{TFp}=\{\begin{array}{c} 1\\ 10\sim 100\\ 0 \end{array}\begin{array}{c} \text{no}\;\;\text{interaction }\\ \text{activation}\\ \text{repression} \end{array}$

A TF can serve as either an activator or a repressor, or simply does not interact with the RNAP, represented by different *w_TFp_* (Table 3). If *w* is set to 1, it represents that RNAP and the TF bind independently to the promoter. If *w* is set to 10~100, it represents that the TF helps to recruit RNAP to the promoter. The larger *w* is the higher the synergism is. If *w* is set to 0 or close to 0, it represents that the TF blocks the RNAP binding, and thus the TF is a repressor.

**Table 4:** The multinomial distribution of a promoter with its RNAP and two regulatory TFs.

**(TF_1_, TF_2_) RNAP**	**(0, 0)**	**(1, 0)**	**(0, 1)**	**(1, 1)**
**0**	1	*q_TF1_*	*q_TF2_*	*w_TF1TF2_ q_TF1_ q_TF2_*
**1**	*q_p_*	*w_TF1 p_ q_TF1_ q_p_*	*w_TF2 p_ q_TF2_ q_p_*	(*w_TF1 p_+ w_TF2 p_*)* w_TF1TF2_ q_TF1_ q_TF2_ q_p_*

The state of the promoter is represented by the row name and the column name. For example, the first row and the second column has RNAP=0 and (TF_1_, TF_2_)=(1,0), which means the promoter is bound by TF_1 _only. The quantity in a cell represents the ratio between the probability of a particular state and the probability of the base state (no RNAP binding and no TF binding).

Similar expressions can be derived for genes with two regulatory TFs capable of binding to a promoter together with RNAP (Table 4). The parameter *w_TF1TF2_* is used to simulate the interaction between the two TFs. A large *w_TF1TF2_* (10~100) represents that the two TFs stabilize each other onto the promoter. If the two TFs have no interaction, *w_TF1TF2_* should be set to 1. If the two TFs compete for the binding, *w_TF1TF2_* should be set to 0 or close to 0. The other two parameters, *w_TF1 p_* and *w_TF2 p_*, represent the interaction between each TF and RNAP, respectively. They can be set to reflect the nature of these interactions similar to *w_TF1TF2._*

The marginal probability of RNAP binding to the promoter is:

$$P[RNAP_{binding}]=\frac{q_{p}+w_{TF1p}q_{p}q_{TF1}+w_{TF2p}q_{p}q_{TF2}+\left(w_{pTF1}+w_{pTF2}\right)w_{TF1TF2}q_{TF1}q_{TF2}q_{p}}{1+q_{TF1}+q_{TF2}+wq_{TF1}q_{TF2}+q_{p}+w_{TF1p}q_{p}q_{TF1}+w_{pTF2}q_{p}q_{TF2}+\left(w_{pTF1}+w_{pTF2}\right)w_{TF1TF2}q_{TF1}q_{TF2}q_{p}}$$

By adjusting the parameters *w_TF1 p_*, *w_TF2 p_* and *w_TF1TF2_*, we can obtain an analytical form for the probability of RNAP binding under different forms of interactions among RNAP and the two TFs. Figure 3 summarizes the parameter choices for two forms of simple interactions and six forms of three-way interactions.

### Linking TF concentration to the probability of promoter occupancy

In this section we describe the influence of TF concentration on the probability of TF binding to the promoter of its target gene. In other words, we seek a function *f* such that *q_TF_=f([TF]).* This function will be used to predict changes in the transcription rate upon changes in TF concentration. Let TF−DNA represent the promoter bound by TF, and the binding process can be denoted as:

TF+DNA→TF−DNA

At equilibrium the concentrations of the substrates are described using the Hill equation: $P(TF_{binding})=\frac{{\left[TF\right]}^{n}}{{\left[TF\right]}^{n}+{\left[K_{TF}\right]}^{n}}=\frac{([TF]/K_{TF}]{)}^{n}}{([TF]/K_{TF}]{)}^{n}+1},$ Where *K_TF_* is the concentration required for half of the TF binding to the promoter and n is the Hill coefficient.

Recall the percentage of promoter bound by TF can also be described using *q_TF_*, the ratio of the probabilities of the promoter in the bound and free states,

$$P(TFbinding)=\frac{([TF]/K_{TF}]{)}^{n}}{([TF]/K_{TF}]{)}^{n}+1}=\frac{q_{TF}}{q_{TF}+1}$$

Therefore, we can obtain: $q_{TF}={\left(\frac{\left[TF\right]}{K_{TF}}\right)}^{n}$

We use the unit of [*TF*] and *K_TF_* as the number of TFs per cell. There have been a few efforts to estimate *K_TF_* from empirical data [33]. In this study, we assume at each time point in the time course, [*TF*] is linearly related to the expression level of the TF, as did in earlier module network studies [34]. It follows that [*TF*] peaks at the same time as its gene expression peaks. We further assume *q_TF_* is maximized at the maximum [*TF*] (see sensitivity analysis for further discussion on this assumption). In this study, we assume that *K_TF_* equates the maximum [*TF*] and it is a linear transformation of the maximum expression value of the gene coding this TF. We adopt the value 1/20 for *q_p_* from Buchler et al [1-3].

### A kinetic model for the quantity of the mRNA of the target gene

Assuming the expression level of a gene is proportional to the probability of RNAP binding to its promoter [1-3], we use a differential equation to model the dynamic changes in RNA expression level.

$$\frac{dG}{dt}=K_{g}(\mathrm{Pr}(RNAP_{Binding}))-K_{d}G$$

, where *G* is the concentration (number per cell) of the transcript; *K_g_* is the maximum number of transcripts synthesized per minute per cell and *K_d_* is the degradation rate of transcripts (per minute per cell).

The maximum rate of mRNA synthesis rate has been estimated to be about one mRNA per 6-8 seconds [35]. Following previous estimates [36,37], we assume that the rate of degradation around 1/6 of the maximum transcription rate. Therefore, we use *K_g_* =10 counts per minute and *K_d_* =10/6 counts per minute in this study.

### Test with synthetic data

As a proof of principle, we first use synthetic data to show the validity of the method. We choose three commonly seen regulatory patterns (Figure 1). These regulatory patterns are: 1. a target gene is activated by one TF (Model 2 in Figure 3); 2. RNAP is blocked by a TF (repressor), and this TF is stabilized to DNA by a helper TF (Model 4 in Figure 3); 3. a target gene is regulated by two interacting activators (Model 7 in Figure 3), and one of the two activators is transcriptionally repressed by a third TF. For each of these three regulatory patterns, we do simulations as follows. First, we simulate the concentration change of each TF over time, which we call real_TF_exp, using equation: E_A_ = a_A_+b_A_log t +ε, where a_A_ and b_A_ are background gene expression index and coefficient describing changes of expression index with time. The ε represents the variability of expression index for gene A. Different patterns of transcription factor expression can be obtained by using different parameters of a_A_, b_A_ and ε. Assuming that the concentration of TF is a linear transformation of E_A_, we feed these simulated concentrations of the TFs into a chosen regulatory pattern described in Figure 1 and derive the expression pattern of the target gene (real_target_exp) according to equations 1 and 2. Noises (normal(0,1)) are added to all the real expression patterns for both TFs and the target gene. We assume only the noise-added expression patterns are observed, and we denote the observed expression values as obs_TF_exp and obs_target_exp. The obs_TF_exp for all TFs in consideration are used to derive expression pattern for the target gene under each model in Figure 3. The model derived expression patterns are termed model_target_exp. For each model, obs_target_exp is compared to model_target_exp in terms of Pearson correlation. To test the robustness of the model, we have assessed the effects of choices of parameters on method performance (see Sensitivity analysis).

### Model fitting

For each target gene, we identify its TF from either literature survey or ChIP-chip data. In this study we focus on genes regulated by two key transcription factors in ESCs: Oct4 and Nanog [13]. For each interaction form in Figure 3, we use the differential equation (eq. 1) to simulate the steady state level of mRNA expression level using a) the estimated [TF] and *K_TF_* based on measured mRNA levels. We simulate a series of steady state mRNA concentrations corresponding to measured expression profile of the target gene. We then compute the Pearson correlation between the simulated concentrations of target genes over time and the observed concentrations from the time course microarray data. The interaction form that predicts a concentration dynamics with a largest correlation to the measured expression level is regarded as the model-identified interaction form.

## Competing interests

The authors declare that they have no competing interests.

## Authors' contributions

XGZ and SZ initiated and directed the project. CCC implemented the algorithm and performed data analysis. CCC, XGZ and SZ wrote the paper.  All authors approved the final manuscript.
